# Tele-Rehabilitation Strategies for a Patient With Post-stroke Spasticity: A Powerful Tool Amid the COVID-19 Pandemic

**DOI:** 10.7759/cureus.19201

**Published:** 2021-11-02

**Authors:** Emanuela E Mihai, Marius N Popescu, Cristina Beiu, Luca Gheorghe, Mihai Berteanu

**Affiliations:** 1 Physical Medicine and Rehabilitation, Carol Davila University of Medicine and Pharmacy, Bucharest, ROU; 2 Dermatology, Carol Davila University of Medicine and Pharmacy, Bucharest, ROU; 3 Physical Medicine and Rehabilitation, Elias Emergency University Hospital, Bucharest, ROU

**Keywords:** physical therapy, spasticity, stroke, tele-rehabilitation, covid-19

## Abstract

During the COVID-19 pandemic, stroke remains a leading cause of death and disability. Since the approach towards patients, treatments, and follow-up regimens has changed, tele-rehabilitation became a pillar for patients with ongoing recovery processes and rehabilitation strategies, especially for post-stroke patients. We describe the case of a 50-year-old male, suffering from right spastic hemiplegia and patellar clonus, for whom rehabilitation services were delivered both in‐person (conventional physical therapy and radial extracorporeal shock wave therapy) as well as with the aid of a tele-rehabilitation program. Assessments were conducted remotely via a self-adapted treadmill and stabilometric equipment, both connected to the Internet. At discharge and at 20 weeks follow-up, the patient showed decreased spasticity grade, improvement in sensorimotor function, balance, functional mobility, clonus score, ambulation distance, and decreased pain intensity. The case highlights the utility of tele-rehabilitation strategies in complementing and enhancing the effects of conventional physical therapy and radial extracorporeal shock wave therapy (rESWT) in post-stroke spasticity.

## Introduction

Stroke is one of the main causes of death and disability in adults worldwide [1-2]. Accordingly, adapted therapeutic interventions and assessment tools should be used from the early phase of stroke, allowing a good risk stratification and timely implementation of effective rehabilitation programs. The rehabilitation process is complex and requires a large amount of time, especially for post-stroke patients. However, since the COVID-19 pandemic, this process and long-term assessments for neurorehabilitation programs became difficult at times. Therefore, tele-assessment and tele-rehabilitation became reasonable alternatives both for practitioners and patients as they could offer an insight into the progress and the maintenance of an adapted level of activity [3-4]. Due to the coronavirus disease 2019 (COVID-19) pandemic, a large number of consultations had to be postponed or delivered through tele-medicine, especially for conditions causing long-term disability [3-4]. Furthermore, the use of telemedicine, tele-rehabilitation, and tele-assessment in everyday practice could help improve the quality of care for persons with chronic afflictions.

We present the case of a 50-year-old post-stroke male for whom the integration of a tele-rehabilitation program proved its effectiveness for adapting and optimizing the neurorehabilitation strategies and therapies used during hospitalization, namely conventional physical therapy and radial extracorporeal shock wave therapy (rESWT).

## Case presentation

The patient consented to participate in this case report, which was conducted in accordance with the Declaration of Helsinki.

A 50-year-old male with post-stroke spasticity and right hemiparesis due to an ischemic stroke was transferred from the Neurology Unit to our Physical and Rehabilitation Medicine Department for the initiation of a neurorehabilitation program. At admission, according to the ratings criteria of the Modified Ashworth Scales (MAS), the patient suffered from grade three spasticity in the right upper limb and grade two spasticity in the right lower limb. Clinical examination also showed a right-sided hemiparesis, hemihypesthesia and patellar clonus. Biologic investigations depicted a mild inflammatory syndrome and moderate anaemia. Based on the clinical and paraclinical grounds, we initiated a conventional physical therapy program combined with rESWT. We define conventional physical therapy as any rehabilitation therapy and technique performed according to each rehabilitation centre or department. Therefore, therapies involving interventions for post-stroke patients (active and passive range of motion exercises, stretching and core stability exercises, stance, balance and gait training, functional training, physical agents, orthoses, etc.) are the components of the conventional physical therapy program. The rESWT (Endopuls 811; Enraf-Nonius B.V., Rotterdam, Netherlands) was applied as follows: 2000 shots with a frequency of 10 Hz, and energy density of 60 mJ (1 bar). The intervention site was the myotendinous junction of the gastrocnemius and the soleus muscles. The patient underwent two rESWT sessions once a week for two weeks.

Clinical and stabilometric assessments were performed at baseline (T0), at the end of the rehabilitation program and after the second rESWT session (T1), and 20 weeks after patient discharge (T2). Overall, the clinical outcome measures showed significant improvement at different assessment times, proving long-term effectiveness of rESWT as a non-invasive treatment for post-stroke patients with spasticity, clonus, pain, mobility, balance, functionality, and gait deficits. Due to the COVID-19 pandemic, the patient could not benefit from a new adapted neurorehabilitation program, including rESWT sessions, botulinum toxin type A injections (BoNT-A), or assessments implying hospital stay but, to ensure the continuity of the rehabilitation plan, tele-rehabilitation sessions with a physical therapist were initiated after discharge. Prior to discharge, the patient was provided with a custom-tailored rehabilitative exercise program and properly instructed about both the clinical and virtual assessments that were to be conducted.

Clinical assessments

The spasticity grade decreased by one point for the MAS, and the effect was maintained at 20 weeks follow-up. Also, for knee and ankle passive range of motion (PROM), the gain in terms of degrees was maintained at 20 weeks after discharge (T2) in comparison to T0, even though it showed a small decrease at T2 compared to the assessment at T1. For the pain intensity assessment by Visual Analogue Scale (VAS), the score decreased by two points at T1 and T2. Concerning the clonus score, it decreased by two points and maintained the same level at T1 and T2. The patient also showed significantly improved mobility, functionality, balance, gait, ambulation speed and safety even in the long term. The Tinetti Assessment Tool, Functional and Ambulation Categories (FAC), Fugl-Meyer Assessment for Lower Extremity (FMA-LE), and Timed Up and Go Test (TUG) were significantly improved. Additionally, TUG scored better results at long-term follow-up, and functional mobility improved compared to the initial and the second assessment. A complete review is provided in Table 1.

**Table 1:** Clinical outcome measures at T0, T1, T2 MAS: Modified Ashworth Scale; PROM: passive range of motion; VAS: Visual Analogue Scale; FAC: Functional Ambulation Categories; FMA-LE: Fugl-Meyer Assessment for Lower /extremity; TUG: Timed Up and Go test. T0: baseline assessment; T1: assessment at the end of the rehabilitation program and the second radial extracorporeal shock wave therapy (rESWT) session; T2: assessment at 20 weeks after discharge.

Clinical outcome measures	T0	T1	T2
MAS	2	1	1
Knee PROM (degrees)	119	125	123
Ankle PROM (degrees)	44	48	47
VAS	3	1	1
Clonus score	4	2	2
Tinetti Assessment Tool	14	23	23
FAC	5	6	6
FMA-LE	22	25	25
TUG (seconds)	31.01	25.04	19.08

Stabilometric assessments

The stabilometric assessment and analysis were carried out by using ProKin 252 (TecnoBody®, Bergamo, Italy) and Walker View system (TecnoBody®, Bergamo, Italy). The ProKin system incorporates a force-sensitive stabilometric platform that provides the evaluation of trunk, stance, static and dynamic balance, and the Walker View system assists with a full postural and walking analysis. The two systems provide real-time visual feedback, which facilitates the improvement of stance during static, dynamic balance, and walking. As for the stabilometric outcomes assessed through the ProKin system, the results were consistent with the clinical parameters, presenting satisfying short-term and long-term effects of rESWT sessions during conventional physiotherapy programs and complemented through tele-rehabilitation strategies. Dynamic balance, trunk analysis, and limits of stability were much improved at T1 compared to the initial assessment and continued to show amelioration at 20 weeks, correlating with clinical parameters. Regarding static balance with eyes-open (EO) and eyes-closed (EC), all parameters scored improved results at T2 compared to T1 and T0, except perimeter with EC in the static balance test. However, the improvement was significant compared to T0 and scored only a mild difference compared to T2. A complete review is provided in Table 2. For Walker View analysis, right foot pitch and right foot roll scored improvements at T1 and T2 compared to T0.

**Table 2:** Stabilometric outcome measures at T0, T1, T2 EC: eyes-closed; EO: eyes-open. T0: baseline assessment; T1: assessment at the end of the rehabilitation program and the second radial extracorporeal shock wave therapy (rESWT) session; T2: assessment at 20 weeks after discharge.

ProKin (Stabilometric outcome measures)	T0	T1	T2
Dynamic	7.59	4.85	3.75
Trunk	359.72	677.07	670.98
Limits of stability	28.92	39.51	38.01
Static-perimeter, mm (EO)	540.58	369.59	320.41
Static-ellipse area, mm^2 (EO)	501.61	311.42	300.01
Static-perimeter, mm (EC)	830.99	570.89	579.98
Static-ellipse area, mm^2 (EC)	1071.46	665.6	601.89

**Figure 1:**
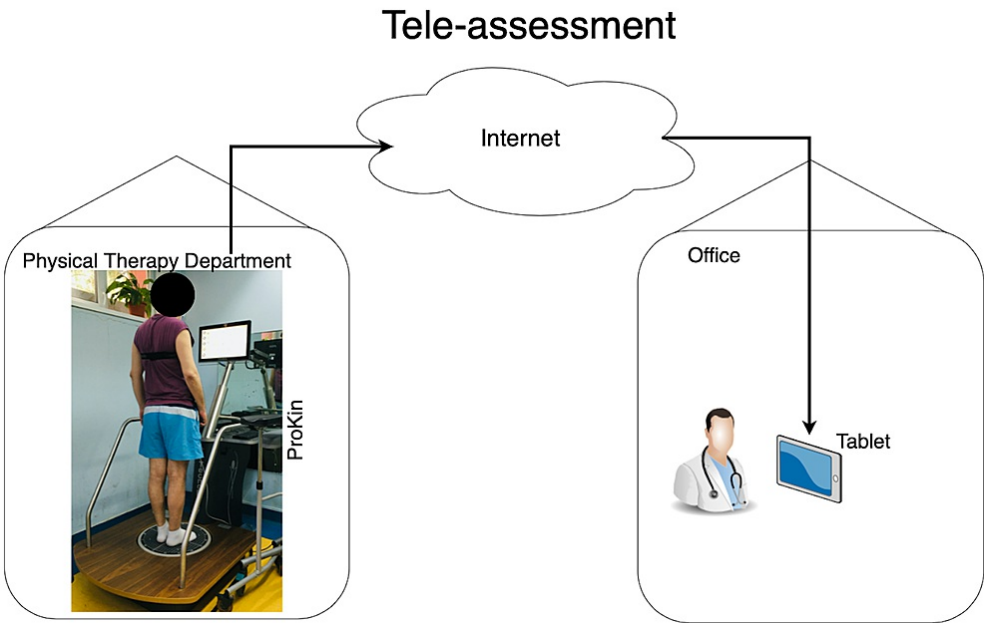
Tele-assessment of stabilometric outcome measures through ProKin system. [Original figure by author Emanuela E. Mihai. The clinical image in the figure was taken in the Department of Physical and Rehabilitation Medicine of Emergency University Hospital (Elias), Bucharest, using a digital camera (Nikon D3300; Nikon Corporation, Tokyo, Japan)]

**Figure 2:**
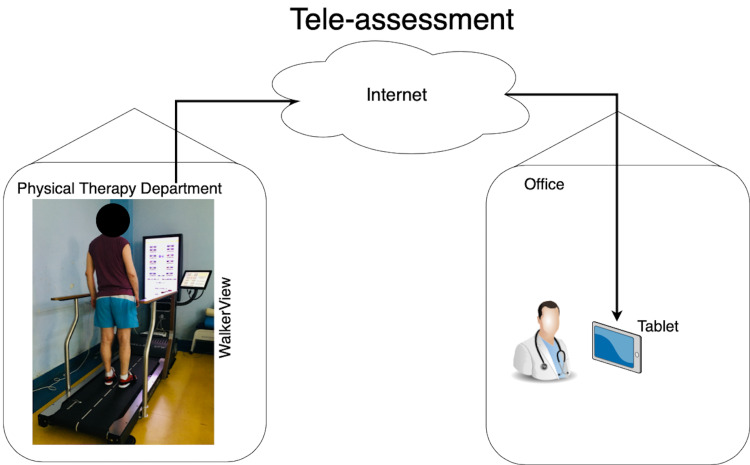
Tele-assessment through Walker View system [Original figure by author Emanuela E. Mihai. The clinical image in the figure was taken in the Department of Physical and Rehabilitation Medicine of Emergency University Hospital (Elias), Bucharest, using a digital camera (Nikon D3300; Nikon Corporation, Tokyo, Japan)]

After the stabilometric assessment was performed by a skilled physical therapist, the results were transmitted automatically via the internet to the assessor’s tablet, which integrated the stabilometric outcome measures with the clinical evaluation, as presented in Figure 1 and Figure 2. We used this form of tele-assessment during the COVID-19 pandemic to ensure a faster, more effective way in which the clinical and the stabilometric parameters can be correlated, and to also assist the patient in every step of the rehabilitation program.

Tele-rehabilitation helped maintain and further improve the results obtained during the hospital stay. At discharge, the patient demonstrated improved physical functional independence with satisfying functional mobility, decreased spasticity grade, reduced clonus and pain symptoms. Ambulation speed, precision, and safety during gait were all improved. At 20 weeks follow-up, after the weekly tele-rehabilitation program, these parameters persisted within the same limits, and the patient also scored better results for TUG, ambulating with more reassurance, mobility, and precision. No adverse events were reported during or after the rehabilitation program. No falls were registered either during the assessments, tests, or hospital stays.

## Discussion

The aim of this case report was to highlight the utility of an adapted tele-assessment and tele-rehabilitation strategy for a recovering patient who also underwent conventional physical therapy and rESWT during the hospital stay. During the COVID-19 pandemic, tele-assessment and tele-rehabilitation techniques were delivered for rehabilitation plan progression and real-time results, providing an objective evaluation in correlation with clinical parameters. Telemedicine for chronic neurological conditions could become part of everyday practice both for practitioners and patients, not only during the COVID-19 pandemic but also in less challenging times [5]. For many patients with chronic afflictions, access to timely treatment became a great issue during pandemic times, and healthcare plans should be adapted [6]. Recommendations should focus on regular physical therapy programs at home, as tele-rehabilitation programs showed their utility.

The patient presented significant improvement of all outcome measures on both short and long-term, with the aid of effective tele-assessment and tele-rehabilitation strategies. The static and dynamic balance were improved, as well as the Tinetti Assessment Tool score. In the eyes-open (EO) setting, area and perimeter decreased significantly, and the results were maintained at 20 weeks follow-up. The only stabilometric parameter that showed few changes in score was the static assessment in the eyes-closed (EC) setting. However, it showed improvement at T2 compared to T0. The clinical improvement of MAS grade showed the effectiveness of the rESWT in the short and long term, and the results are consistent with those from various studies [7-8]. Knee and ankle PROM showed significant improvement after the treatment. Several studies have already presented satisfying effects of rESWT therapy on post-stroke spasticity [7-8]. Additionally, rESWT also reduced pain intensity and clonus score through its effects on the muscles and tendons, providing long-term tissue regeneration, antalgic and anti-inflammatory properties [8-9]. FMA-LE showed sensorimotor and functional improvement in correlation to the stabilometric outcomes. However, for a cumulative effect, BoNT-A injections [10] and other injectable biomaterial-based therapies commonly used in regenerative medicine [11] could represent viable complementary therapies. 

During the COVID-19 pandemic, the follow-up process of post-stroke patients was highly affected. To promote the rehabilitation continuity and reassessment of all outcome measures, we proposed a tele-assessment and tele-rehabilitation approach. Through this approach, we were able to further reassess spasticity grade, clonus, pain intensity, balance, mobility, functional capacity, and gait and to correlate the new findings with the already collected data during the hospital stay. Integrating clinical examination and virtual assessment provided both the patient and the rehabilitation team with objective data by tracking progression landmarks, further leading to a more adapted, individualized therapeutic approach.

Another extremely important aspect is that classic rehabilitation programs for stroke survivors imply high costs, thus representing a huge burden on healthcare systems worldwide, especially in low-and-middle income countries. In the management of post-stroke patients, tele-rehabilitation was shown to be cost-effective when compared to in-clinic intervention [12]. It also allows access to a rehabilitation program for patients who cannot otherwise receive proper care, thus highly increasing patient satisfaction from this perspective. Future research on the efficacy and cost-effectiveness of tele-rehabilitation in post-stroke patients is needed.

## Conclusions

The case highlights the usefulness of tele-rehabilitation and tele-assessment in enhancing the effects of conventional physical therapy and rESWT in post-stroke spasticity. This strategy was essential for our patient during the pandemic when access of post-stroke patients to their usual in-clinic rehabilitation program and follow-up regimen is limited. The patient presented improved lower limb spasticity grade and trunk control, decreased pain intensity and clonus score, improved static and dynamic balance, enhanced sensorimotor performance, functionality, and gait. Through tele-rehabilitation sessions, treatment effectiveness lasted up to 20 weeks, showing that tele-rehabilitation programs are key factors for both practitioners and patients, especially in these unprecedented COVID-19 times. In addition, rESWT showed a good safety profile and long-term efficacy for all the outcome measures. Future research is needed concerning adapted approaches of tele-rehabilitation techniques, therapies, and assessment tools.
